# Editorial: The Impact of Stress on Cognition and Motivation

**DOI:** 10.3389/fnbeh.2018.00326

**Published:** 2018-12-21

**Authors:** Pedro Morgado, João J. Cerqueira

**Affiliations:** ^1^Life and Health Sciences Research Institute (ICVS), School of Medicine, University of Minho, Braga, Portugal; ^2^ICVS-3Bs PT Government Associate Laboratory, Guimarães, Portugal

**Keywords:** cognition, stress, motivation, behavior, decision-making

Stress is usually defined as an actual or anticipated threat or disruption of organism homeostasis, which usually leads to an acute stress response allowing for adaptation to the new condition. Conversely, chronic stress usually leads to maladaptive responses in various organs and systems, activating pathophysiological mechanisms such as psychiatric disturbances, neurologic disorders, cardiovascular diseases and metabolic dysregulation (Sousa, 2016). The impact of chronic stress in cognition and motivation has been extensively described in the literature. In this Research Topic of Frontiers in Behavioral Neuroscience, a host of new empirical findings on the impact of stress on cognition and motivation was presented in a translational perspective from rodents to humans.

The detrimental impact of stress on behavior can be established early in development, including from exposure before birth. Usually described as programming effects, these are well described after strongly stressful experiences during pregnancy. In one of the papers of this series, Soares-Cunha and co-workers extend our understanding of programming effects by showing that even mild stressful events during this critical period can lead to long-lasting structural and neurochemical alterations and anxious- and depressive-like behaviors in the offspring (Soares-Cunha et al.).

Social stress is also known to induce depressive-like behaviors. Using tests based on association learning, Kúkel'ová et al. show that chronic social stress (CSS) can lead to reductions in reward salience and effort valuation in mice, decision-making deficits that can also be observed in patients depression and schizophrenia (Kúkel'ová et al.), adding to the external validity of the model. Moreover, in another paper of the series, by Yang and collaborators, CSS induced spatial-working and contextual-fear memory deficits were related with decreased levels of N-methyl-D-aspartate receptor subunit 2B (NR2B), impaired long term potentiation (LTP) and NMDA receptor-mediated excitatory postsynaptic currents in the hippocampus (Yang et al.). Importantly, both memory impairments and electrophysiological alterations were attenuated by antidepressant treatment with the NMDA-antagonist ketamine (Yang et al.), adding further evidence to support a role for glutamate excitotoxicity in such stress-related deficits.

Although post-traumatic stress disorder (PTSD) is a widely recognized and often devastating consequence of exposure to intense stress, the biochemical mechanisms underlying the creation of the fear memory remain poorly understood. In a very interesting paper in this regard, Han and co-workers show that the behavioral consequences of exposure to a stress protocol designed to mimic a PTSD like-condition [single prolonged stress (SPS) protocol and immobilization-stress (IM)] are associated with decreased LTP as well as decreased stathmin and increased Rin1 expression in the hippocampus and the amygdala (Han et al.). Of note, both regions are implicated in the regulation of fear memory, further stressing the significance of these findings.

The serotoninergic system has been involved in both anxiety state and trait, particularly through the activity of 5-HT2A receptors. To assess how anxiety trait can modulate the role of 5-HT2A in stress-induced anxiety, León and collaborators treated animals with high and low freezing responses to contextual cues previously associated with footshock with ketanserin, a preferential 5-HT2A receptor blocker. Their finding of an opposite effect of the drug on the two lines highlights and adds insight into the relevance of genetic variability in the establishment of stress responses (León et al.).

Like serotonin, glial-derived neurotrophic factor (GDNF) has been implicated in the regulation of stress responses. Specifically, it was previously recognized that up-regulation of GDNF expression is associated with increased stress resilience. In a paper in the present Research Topic, Buhusi and co-authors extend this findings by showing that the opposite is also true: GDNF-deficient mice are more vulnerable to stress, failing to express latent inhibition (LI) which results in slower learning of new conditioning associations (Buhusi et al.). Moreover, this LI impairment was associated with a decreased neuronal activation in the nucleus accumbens shell and increased activation in the nucleus accumbens core.

Decision-making processes are among the cognitive-related dimensions widely affected by stress. Work from ours and other groups has shown that chronic stress promotes a switch from a flexible and contextualized goal directed system of responses to a more rigid habit based system (Dias-Ferreira et al., 2009). In line with this, a paper by Maran and colleagues in the present series shows that high arousal states disturb spatial and sequence learning, discrimination of spatial positions and learning of associative sequences, which could all reflect a reduced involvement of the flexible cognitive systems responsible for the sensitivity for contextual details (Maran et al.).

We have previously shown that risk-based decision making is also impacted by chronic stress exposure (Morgado et al., 2015). In the present Research Topic, Simonovic and colleagues translate our findings to humans, showing that stress exposure impairs performance in the Iowa Gambling Task (IGT), delaying the avoidance of the disadvantageous decks (Simonovic et al.). In the same vein, Starcke and colleagues show that the exposure to unsolvable anagrams also induces more disadvantageous decisions on the IGT (Starcke et al.), an effect particularly observed in male participants.

Given the vast consequences of stress exposure and its ubiquitous presence in our everyday lives, research on attenuating measures is of high clinical and even societal relevance. In a very interesting paper of this series, DiMenichi and co-authors show that writing about past failures before experiencing a stressor attenuated subsequent stress responses and reduced their physiological and behavioral effects (DiMenichi et al.), a finding that can be easily transposed to practice.

Altogether, the findings put forward in this research topic contribute to a better understanding of how stress impacts on cognition and motivation, providing a broad range of insights from the molecular and cellular processes that underlie behavioral alterations to new interventions that can ameliorate stress-induced impairments.

## Author Contributions

PM and JJC wrote this article and approved it for publication.

### Conflict of Interest Statement

The authors declare that the research was conducted in the absence of any commercial or financial relationships that could be construed as a potential conflict of interest.

## References

[B1] Dias-Ferreira E. Sousa J. C. Melo I. Morgado P. Mesquita A. R. Cerqueira J. J.. (2009). Chronic stress causes frontostriatal reorganization and affects decision-making. Science 325, 621–625. 10.1126/science.1171203 19644122

[B2] Morgado P. Marques F. Ribeiro B. Leite-Almeida H. Pego J. M. Rodrigues A. J.. (2015). Stress induced risk-aversion is reverted by D2/D3 agonist in the rat. Eur. Neuropsychopharmacol. 25, 1744–1752. 10.1016/j.euroneuro.2015.07.003 26233608

[B3] Sousa N. (2016). The dynamics of the stress neuromatrix. Mol. Psychiatry 21, 302–312. 10.1038/mp.2015.196 26754952 PMC4759204

